# Inequities in access to directly-funded home care in Canada: a privilege only afforded to some

**DOI:** 10.1186/s12913-023-09048-9

**Published:** 2023-01-18

**Authors:** Christine Kelly, Lisette Dansereau, Maggie FitzGerald, Yeonjung Lee, Allison Williams

**Affiliations:** 1 Department of Community Health Sciences, University of Manitoba, Winnipeg, MB Canada; 2 Department of Political Studies, University of Saskatchewan, Saskatoon, SK Canada; 3 Social Welfare, Chung-Ang University, Seoul, South Korea; 4 School of Social Work, University of Calgary, Calgary, AB Canada; 5 School of Earth, Environment & Society, McMaster University, Hamilton, ON Canada

**Keywords:** Inequity, Directly-funded home care, Direct payments, Consumer directed care, Canada

## Abstract

**Background:**

Directly-funded home care (DF) provides government funds to people who need assistance with the activities of daily living, allowing them to arrange their own services. As programs expand globally, many allow DF clients to hire home care agencies to organize their services rather than finding their own workers. In Canada, half of the DF home care programs allow users to purchase agency services. The goal of this research is to describe the role of agency providers in DF home care in Canada and consider potential equity implications for service access from the perspectives of clients and families.

**Methods:**

Framed with intersectionality, the study included online focus groups with families and clients (*n* = 56) in the two Canadian provinces of Alberta and Manitoba between June 2021-April 2022. All transcripts underwent qualitative thematic analysis using open and axial coding techniques. Each transcript was analyzed by two of three possible independent coders using Dedoose qualitative analysis software.

**Results:**

The article presents five thematic findings. First, the focus groups document high rates of satisfaction with the care regardless of whether the client uses agency providers. Second, agency providers mediate some of the administrative barriers and emotional strain of using DF home care, and this is especially important for family caregivers who are working or have additional care responsibilities. Third, there are out-of-pocket expenses reported by most participants, with agency clients describing administrative fees despite lower pay for the frontline care workers. Fourth, agencies are not generally effective for linguistic and/or cultural matching between workers and families. Finally, we find that DF care programs cannot compensate for a limited informal support network.

**Conclusions:**

Clients and families often intentionally choose DF home care after negative experiences with other public service options, yet the results suggest that in some Canadian contexts, DF home care is a privilege only afforded to some. Given the growing inequalities that exist in Canadian society, all public home care options must be open to all who need it, irrespective of ability to pay, degree of social support, or competence in the English language.

**Supplementary Information:**

The online version contains supplementary material available at 10.1186/s12913-023-09048-9.

## Background

Directly-funded home care (DF) provides government funds to older people, people with disabilities, and caregivers to arrange their own assistance with the activities of daily living. DF^1^ home care is expanding globally as a potential avenue to relieve strained home care systems [1–5]. Systematic reviews find that clients are more satisfied with DF over other home care options that do not allow for as much input and control [4, 6]. Yet, research also finds that expanding DF programs “can inadvertently extend health inequities” for people with intellectual disabilities and those living in rural settings, posing barriers to arranging care [7]. Another study found that DF reinforces inequities across class and gender, as “those with higher income” are more likely to access the program and men report relying on informal care provided by female partners to supplement the program [8]. Research on direct payments in the United Kingdom has long raised concerns about barriers to access for certain groups such as older people, people with learning disabilities, and people living with mental health issues [9, 10]. There is an urgent need for research that attends to potential health inequities caused by DF policy design and expansion.

One key policy design issue is whether clients should be permitted to use the funds to hire home care agencies. In many contexts, DF programs originated to enable adults with physical disabilities to hire care workers from their personal networks through an individual employer model. Workers often do not have any formal training, and friends and family are sometimes hired [11]. This can allow clients to pursue workers with shared linguistic, cultural and other individual characteristics, although there is limited research on whether this actually happens. One study of diverse stroke survivors found linguistic barriers to initially accessing DF [12]; another study confirms the need to remove linguistic barriers and suggests language-matching is a key facilitator for high quality care for South Asian older people in Britain [13].

As DF programs expand and evolve globally many allow DF funds to be used to hire home care agencies to organize the services, rather than requiring clients to find workers themselves. In Canada, for example, all 10 provinces have a DF home care program and half of them allow clients to use the funds to hire home care agencies [14]. Yet, much of the research establishing the benefits of DF models of care are based on individual employer models in comparison to agency services. The foundational study by Carlson et al. [2] found DF users had “major improvement in their care and overall well-being” as compared to a control group using agency services [2]. Similarly, Benjamin et al. [15] found that DF clients “report more positive outcomes than those in the agency model, or they report no difference.” In both studies, the control groups were clients using agency services. The results are thus not directly applicable to other contexts, such as Canada, where DF is used both to hire agencies and/or to hire workers directly as an alternative to government-organized services.

The goal of this research is to describe the role of agency providers in DF home care in Canada and consider potential equity implications for service access from the perspectives of clients and families. Through in-depth qualitative focus groups with 56 families and clients in two Canadian provinces, we share five thematic results. First, participants express high rates of satisfaction regardless of whether they use agency providers. Second, agency providers mediate some of the administrative barriers and emotional strain of using DF home care, and this is especially important for caregivers who are working or have additional care responsibilities. Third, there are out-of-pocket expenses reported by most participants, with agency clients describing administrative fees despite lower pay for the frontline care workers. Fourth, agencies are generally not effective for linguistic or cultural matching. Finally, we find that agencies cannot compensate for a limited informal support network. Clients and families often choose DF home care after negative experiences with other service options, yet it emerges that in some Canadian contexts, DF home care is a privilege only afforded to some.

### Canadian context

Canada has a federal-provincial/territorial governance structure with home care services falling under provincial/territorial jurisdiction. There is wide variation in the availability, cost, eligibility, and organization of home care services across the country [16, 17]. Canada’s universal health care system is governed by the Canada Health Act – which does not include home care services; nevertheless, all jurisdictions offer some degree of public home care services. Eligibility is based on a clinical assessment of need for assistance with the activities of daily living (ADLs) and sometimes for the instrumental activities of daily living (IADLs) such as housekeeping. Eligibility may be income-based or universal depending on location. Population research documents 41.1% of Canadian households use formal home care services covered by government support; on the other end of the spectrum, 35.4% of households pay entirely out-of-pocket [18]. To further complicate this landscape, home care may be delivered by public employees, by public contracts with for-profit and/or non-profit home care agencies, or a combination.

In terms of DF home care programs specifically, there are options in all 10 provinces but none in the territories. DF home care operates as a niche option serving a small proportion of home care recipients in most locations [14, 17]. Our prior research identified the use of agency providers as a contentious and poorly understood manifestation of Canadian DF home care [19]. There is limited and dispersed information on the number, size, and practices of agencies operating in each province. To better understand this issue, we investigated the role of agency providers in two DF home care programs with a high proportion of agency use in the provinces of Manitoba and Alberta.^2^

### Theoretical framework

The study draws on intersectionality, a theoretical tool and methodology that connects oppression and privilege to lived experiences [20, 21]. A foundational concept in feminist and gender studies, intersectionality is rooted in anti-racist and feminist movements, championed by black feminist activists and scholars such as Kimberlé Crenshaw, the Combahee River Collective and Patricia Hill Collins [20, 22, 23]. Intersectionality demonstrates how lived-experiences are shaped by the complex and shifting interactions of gender, race, class, dis/ability, and other key identity factors and is well-suited to qualitative research [24, 25]. Population and public health research is increasingly drawing on intersectionality to explain effects of social location on health and well-being [26, 27].

## Methods

Data collection included online focus groups with DF home care clients and/or their supports in each province. Focus groups are an ideal method for comparing experiences of groups of people and have been used successfully in studying DF programs in other countries [28, 29]. Focus groups were held using Zoom between June 2021-April 2022. Remote data collection was used in light of the Covid-19 pandemic.

DF clients and families were recruited in two regional health areas based in large metropolitan cities in the provinces of Manitoba and Alberta. The health region in Alberta was selected due to a pilot DF program designed for clients who want to hire agencies. At the time of the study, the Alberta health region had 704 clients enrolled in DF (including 216 on the pilot program). The Manitoba health region was selected due to a high proportion of agency use (40% of clients), with approximately 980 total DF clients at the time of the study.

Participants were recruited through an information package emailed or mailed to all clients in Alberta and clients with an active email address in Manitoba (physical mail out was not feasible at the time due to pandemic strain). Recruitment was facilitated by government collaborators using a single blind recruitment strategy so that the research team did not have access to personal information of the clients unless they chose to contact us. Potential participants were directed to a secure research website where they completed preliminary screening questions confirming their province of residence, enrollment in a relevant DF program, and their current hiring mechanism. If eligible, potential participants read and could elect to sign an online consent form, provide basic demographic information, and indicate preferred dates and times for focus groups. In line with our intersectional framework we prioritized the participation of multiply-marginalized individuals by re-scheduling when necessary. If language or computer access was an issue, interested participants could enroll by phone.

There were seven groups in Manitoba, with 32 participants in total, and seven groups in Alberta, with 24 participants, making for a total of 56 participants. The study took place during various waves of the pandemic and some of the resulting groups had very few participants. Qualitative methods research finds four focus groups of 6-10 participants per relevant group stratification are usually sufficient for thematic coding saturation; [30] we adjusted the participant targets based on the assumption that focus groups typically include at least six participants and aimed for at least 24 participants in each province and/or hiring mechanism. The focus groups were led by the first author (C. Kelly) or the second author (L. Dansereau) with two student research assistants taking notes. All participants were offered an honorarium or gift card ($25 CAD) in line with established research practices [31].

All groups had rich responses, even those with few participants. Participants were asked about their home care experiences and decisions, individual situations, with questions designed to facilitate intersectional analysis (see Additional file 1 Appendix 1: Focus group guide). Client demographics are summarized in Table 1. At the time of the focus group, 21 participants were hiring agencies, 33 were acting as individual employers, and 2 participants were doing both. Notably, 14 participants (25%) had experiences with both approaches and their participation greatly strengthened and confirmed thematic findings. Most of the participants were born in Canada (68%), were financially comfortable (75%), were white (71%), women (61%), and between the ages of 45 and 80.

**Table 1:** Focus group participant demographics (*n* = 56)

Descriptor	Category	Participants	Proportion
**Age**	25–44	7	13%
	45–64	26	46%
	65–80	23	41%
**Gender**	Woman	34	61%
	Man	22	39%
	Non-binary or other gender identity	0	0%
**Ethnicity**	Person of colour	10	18%
	White	40	71%
	Prefer not to answer	6	11%
**Country of birth**	Born in Canada	38	68%
	Born outside of Canada	17	30%
	Prefer not to answer	1	2%
**Financial wellbeing**	Never have trouble	42	75%
	Sometimes have trouble	6	11%
	Often have trouble	3	5%
	Prefer not to answer	5	9%
**Hiring mechanism**	Individual employer	33	59%
	Purchase from an agency	21	38%
	Both	2	4%
	Experience with opposite hiring mechanism	14	25%

All focus groups were professionally transcribed and underwent qualitative thematic analysis using open and axial coding techniques and Dedoose qualitative analysis software [32–34]. To account for the variation in focus group size, the coding focused on individual responses rather than group discussion. Each transcript was analyzed by two of three possible independent coders [Christine Kelly, Lisette Dansereau, and a student research assistant]. The coding structure was determined through regular discussion [24, 33–36]. In order to apply the theoretical framework, participant details such as gender and individual circumstance are included in all quotations and we report on a non-dominant theme shared by participants from ethnic and cultural minority groups. Intersectionality posits that privilege can shift depending on the circumstance—as such, some themes emerge as commonly shared and others differing when considering social location. See Table 2 for information on the codes used for this article. Participants are given pseudonyms in all publications.

**Table 2:** Main themes and associated qualitative codes

Theme	Sub-theme
1 High satisfaction, regardless of arrangement	Other care experiences
	Reasons for enrolling in DF home care
2 Agencies mediate administrative burden	Agency hire
	Agency advantages
3 Out-of-pocket expenses	Agency hire
	Out-of-pocket expenses
4 Linguistic and/or cultural matching	Intersectionality: culture and ethnicity
	Intersectionality: language
	Intersectionality: religion
5 Informal support networks	Social networks and informal supports
	Living arrangements

## Results

There are five key themes that emerged from the focus groups related to agency use and DF home care: (1) high satisfaction, regardless of arrangement; (2) agencies mediate administrative burden; (3) out-of-pocket expenses; (4) linguistic and/or cultural matching; (5) informal support networks. There were very limited differences in findings across the two study sites.

### Theme 1: High satisfaction, regardless of arrangement

Our focus group discussions found that in most cases, accessing DF home care is an intentional shift away from other government-managed services. That is, moving to DF home care is often a result of *dissatisfaction* with other public services. Lydia, an individual employer for her 91-year-old mother, shares a typical trajectory:

Lydia: We started off actually with [government] home care for about a year and a half and we found that was not working. We were not getting consistent help, the quality of service wasn’t there and everything. So then we thought we need somebody a bit more consistent as [my mother] ages. So we just got onto [DF] care. (Alberta, group 8)

Other reasons cited for regular home care “not working” include inflexibility in tasks the workers can do, rigid scheduling, rushing, and a high absentee rate. For example, in focus group discussions from Manitoba, Drew, an individual employer arranging services for his mother with mild dementia, and Giuseppe, hiring an agency to support his wife with Lewy body dementia, describe similar situations to Lydia when asked why they enrolled in DF:

Drew: My mom is almost 99. We started when she was 97. She had [government] home care coming in and, like the [other focus group participants] said, very erratic. […] So when you take a 97 year old lady and tell her she's got 30 minutes to get out of bed, get dressed, get washed, get down for breakfast… it ain’t going to happen. It was actually our area home care coordinator that agreed with our frustrations said [government] home care is so totally understaffed. And that she suggested we look at [DF]. And we did and what we found is the flexibility is just awesome. (Manitoba, group 3)

Giuseppe: We had [government] services, for approximately a year and there’s a certain randomness to it – which I think others [in this group] have pointed to – that you’re not sure who’s coming and you’re not sure when they’re coming and occasionally you’re not sure if they’re coming…. And we’ve had [DF] care through a company for two years. And it’s much more successful, in terms of the bonding with the person – with my wife – and in terms of the ability to get the supports. (Manitoba, group 2)

Consistently among the participants in this study, families and clients are more satisfied with DF home care especially as compared to government home care services due to the improved consistency and increased flexibility in the timing and types of the workers can do; this satisfaction was not related to whether or not the client and family were using an agency or acting as an individual employer. The thematic analysis did not reveal notable differences in satisfaction linked to intersectional social locations.

### Theme 2: Agencies mediate administrative burden

Among those using an agency, families and clients talked about the ways agencies mediated the administrative burden of using DF home care. Lily, an older spousal manager, described arranging care after her husband had a stroke; unusually among the participants in this study, Lily and her husband went directly on to the DF home care option instead of accessing other services first:

Lily: It happened rather suddenly. And when he was coming home from the hospital, I pursued the different options. […]. And I think [agency] was better for us because I didn't want to have to go through the process of hiring somebody myself. I just didn't have the capacity to go through the posting, recruiting and all of the administration that went along with it. So it just seemed like the best solution to be able to go to an agency, you do the hiring, send me the person that fits the bill. (Alberta, group 14)

Families and clients reported using an agency to help with paperwork, hiring, and back up care. Gina, also an older spousal manager described “I just went for the [agency] option because I have enough on my head without having to worry about finding somebody. I’m new to the area; I don’t know a lot of people and I just wanted someone that was reliable and good” (Alberta, group 12). Lynne, supporting both parents and organizing DF services for her father, said “I don’t have the time at all to deal with hiring – and I don’t have the expertise and that’s why I relied on the agency” (Manitoba, group 6).

One participant, Leah, an older woman with disabilities who was arranging care for herself for a number of years, summed up the first two themes – enrolling in DF home care because of dissatisfaction with other public services and hiring an agency to reduce her administrative burden:

Leah: I had home care for approximately six years. It was unreliable, inconsistent and basically a band-aid. It was just a horror show. […] And same thing, I [switched to DF home care and] went through an agency. Although I was certainly prepared to do the leg work, as far as the – all the issues that had to be done with doing it on your own. But when this agency came up, I thought, no, I’d have to be silly not to take it. And it’s just been a godsend. It’s not perfect, but it’s excellent. (Manitoba, group 6)

Reducing burden was especially important for family managers who were working or had additional care responsibilities (e.g., children). Arlen, caring for his wife and working full-time, commented:

Arlen: [agencies are] not as overwhelming. When [DF home care] was first proposed to me and they started throwing all these things at me, worker’s comp and I had to set up with [payroll taxes] or whatever it was, certain accounts, I can’t even remember, it was very overwhelming. (Manitoba, group 1)

Similarly, Lynne, working full time, supporting both parents and organizing DF home care for her father, describes:

Lynne: We had the option of hiring our own staff. I would say flat out that there was no way having a fulltime job that I was going to be able to hire my own staff and deal with payroll and all of that other stuff. (Manitoba, group 6)

Lily, an older spousal manager quoted previously in this theme, explicitly mentioned that using an agency “alleviates some of the stress. I don't need more stress in my life” (Alberta, group 14).

When focusing on the reasons for using DF home care and satisfaction, a relatively positive picture begins to emerge; however, the narratives reveal diverse and sometimes divergent experiences related to ability to pay out-of-pocket expenses, importance of linguistic and/or cultural matching, and the availability of informal supports. These three factors shaped whether or not using an agency was experienced as advantageous or not.

### Theme three: Out-of-pocket expenses

It was common for families and clients to “top up,” “pay the difference,” or “add hours” when using DF home care – whether through an agency or acting as an individual employer. There were many comments across the focus groups about the additional financial requirements associated with DF home care. Participants expressed being in a financial situation that enabled them to use DF home care with the addition of their own monies. For example, Viola who was herself over the age of 65 and organizing care for her mother-in-law, explains:

Viola: “We’re very lucky, my father-in-law was careful and he left enough so that we were able to hire a lawn service to look after the yard, and we have a cleaning service to come and clean the house for her. So, because they don’t do heavy cleaning. And, I mean, we are in a very lucky situation financially for her.” (Manitoba, group 1)

In the same focus group, Elisha, an older man organizing support for his wife, adds “you do need some more of your own financial resources [to use this program].” The participants who indicated they did not contribute additional funds often identified a specific reason why, for example having a spouse as “free labor” (Alberta, group 10) or because they were eligible for additional funding through other specialized programs or insurance settlements.

There were shared reasons for the out-of-pocket expenses across both groups as participants discussed needing “more care” or additional hours. For example, as Marg said, “Most of the time, well all the time, you don’t get enough hours and so you would have to pull either your own resources or your own funds, your own money” (Alberta, group 9). Yet, there was a clear difference in the reasons provided between the two categories of participants. For those using agencies, participants commonly spoke about the gap between funded hourly rates and the rates charged by agency providers. Travis, an older person living with a progressive neurological condition and arranged care for himself, explains “My [agency’s] rate is 25 bucks [an hour] … And that means that the difference between what the province contributes and what I contribute is a tax-deductible amount of anywhere from $6,000 to $10,000 [per year]” (Manitoba, group 2). Jaime, from the same focus group, also commented on the gap between DF funding and agency rates: “It’s a rate issue. They charge 30 bucks an hour. The system pays $21.40. We make up the difference. We recognize we have the luxury of being able to afford that.” In Alberta the funding rates were higher but nevertheless may not cover agency charges, as reported by Gina, arranging care for her husband with dementia:

Gina: [DF home care] is giving me $31.49 per hour and I am paying $35 per hour … my fear is there’s going to come a time that I am going to need more hours and I don’t know how I’m going to afford it, because it’s still about – the difference is still about 100-something dollars a month and that’s a lot of money. (Alberta, group 12)

Drew described subsidizing care “by about 40%” while in another discussion, Jack commented “reliability, consistency and dedication are priority items for me, and so, you know, we get a little bit from home care, so many hours, but we cover approximately $40,000 a year over and above that for our staff.”​ Marjorie explained that DF funds were not covering the cost of agency services for her mother due to a two-hour minimum booking:

Marjorie: We’re paying out-of-pocket for the difference. We are given I think it’s 20 minutes, 20, half an hour twice a week [in DF home care funds] and the contract with the independent agency is for two hours three times a week. So that’s, it’s a huge difference, which fortunately my mother can afford. (Manitoba, group 7)

For those acting as individual employers, a reason cited for contributing additional funds was to improve working conditions. It is beyond the scope of the article to fully report on themes related to working conditions, yet there was consensus among participants that workers “take home” less pay when hired through an agency as opposed to being employed directly. Many of the participants were concerned about the welfare and working conditions for the workers –and participants were not necessarily aware which agencies were for-profit or nonprofit. For some participants, this awareness factored into their decision to act as an individual employer. For example, Julia, arranging services for her in-laws and previously for her recently deceased mother, described “With going through an agency unfortunately the agency takes a large chunk of what the worker would make. And that fee is really why I wanted to be able to pay the person directly so that $10 an hour of their money really wasn't going to the agency” (Manitoba, group 3). Hao explained switching from an agency to an individual employer “I found that in hiring yourself and also setting the wage yourself, you could enhance the motivation and also you could give them decent pay” (Alberta, group 9).

The costs associated with using an agency contributed to why some of the participants ended up using the individual employer model. Crystal, a spousal care manager working full time, described having to switch agencies due to increases in fees and eventually being forced into the individual employer approach: “Then we switched to another company and then we just kept going. It was the same story over and over. So finally we hire our own people now, we don't go through the agencies anymore” (Manitoba group 5). Robyn, hiring workers to care for her mother, commented “[agencies] are OK for interim plus the cost is, you know, about a third higher than what I would be able to manage; so they’re kind of cost prohibitive” (Alberta, group 11).

Not all of the participants in our study were accessing DF to support them to stay in their own houses, condominiums, or apartments; some of the participants were living in private retirement communities or settings that provided some level of assistance and were using DF to “top up” or supplement this care. These were among the only exceptions of those not paying to top up their DF home care. Indeed, they were already paying extensively for other care arrangements. Rachelle, who moved her husband into a private supportive living environment to avoid him being transferred into residential long-term care home, describes:

Rachelle: I do everything. I’m 77 years old myself. And if I, you know if I got sick or if something happened to me, or there was something, there is no back up. So that’s why this Seniors Residence – Assisted Living – is actually [Laughs] an expensive kind of backup that we’re paying for. (Manitoba, group 2)

In a particularly compelling story, Neve who self-identified as Black African and describes her and her mother as low-income, explained their inability to use the agency option due to the financial barriers related to having to pay for services first and then apply to the local DF program for reimbursement:

Neve: We have to provide the money that is meant for the agencies directly to the agencies and then seven days later home care will reimburse us. Well, I don’t have the $1,000 hanging for – you know, anywhere in my house or anywhere, so – and [the care coordinator] suggested, “Well, get the credit card to –.” No, credit card is for emergencies, lots of emergencies happening out of the blue, I don’t want to be caught without money if something happens. So they said, “Well, you cannot use [an agency] unless you pay the agency first and then we’ll reimburse you.” […]So I say, “Well, then I guess I’ll stay on [as an individual employer]. (Alberta, group 12)

In summary, it was common for participants to contribute personal expenses in order to use DF home care, with differing reasons based on hiring mechanism.

### Theme four: Linguistic and/or cultural matching

In light of our intersectional approach, we share a non-dominant theme from the focus groups that represents the experiences of specific participants from diverse ethnic backgrounds. If a family member had limited fluency in English it was important to find workers with a shared linguistic ability and sometimes shared cultural backgrounds. Agencies were not effective at meeting this need, and participants described choosing the individual employer model to address this. Alexis and her family immigrated from the Ukraine, and she described her reason for being an individual employer: “we need to hire someone who can speak a language that our parents are speaking” (Manitoba, group 1). In another focus group, Lydia, caring for her 91-year-old mother who did not speak English, described a similar reason for choosing the individual employer path: “we needed specifically somebody that could speak Chinese, our dialect of Chinese. So it was really hard that way. But eventually we found someone” (Alberta, group 8). Neve, whose family originated in Eritrea, and Marcia, whose family originated in the Ukraine, both concurred:

Neve: I was able to hire people that speak my mom’s language, Tigrinya, and that they are from the same culture, Eritrean background. […] That was number one for me and it made a big difference. When we went to [an agency for a while], it was harder to find anybody because most agencies do not have people from my country.” (Alberta, group 12)

Marcia: I was able to actually hire my own people who spoke the language and who actually listened to me. […] I'm limited to only people that I know who can speak either Polish or Ukrainian or maybe Russian to him who can help me with care whether it's paid or unpaid.” (Alberta, group 14)

There was only one example in the focus groups of linguistic and cultural matching through an agency; notably, the person was seeking a Filipino caregiver who spoke Tagalog, which is a relatively common immigration group in the two provinces in this study (Manitoba, group 6). In each case described in this theme, the care manager spoke English and was able to arrange DF home care on the care recipient’s behalf; this suggests that those families who do not have an English-speaking member to arrange linguistic matching are less likely to be successful in doing so.

The desire for worker matching was not limited to language, but also involved culture and shared values. Andrzej, who was arranging care for themselves while also holding a professional career, described choosing to become an individual employer:

Andrzej: And I’m a Filipino so my caregivers are all Filipinos. So I can speak my own language.

Interviewer: Have you decided [to hire Filipino workers] specifically because they share your ethnicity?

Andrzej: Yeah and it’s easier. They know how to cook my food, they know that I need to go to church on Sundays. They can come. It’s easier. (Alberta, group 9)

Lydia described a compelling example culturally safe and person-centred care that happened when she was able to match a Chinese worker with her mother:

Lydia:[my mother] doesn’t speak English, so we need someone that speaks the same language, and that can actually cook Chinese food and that will go out to the garden and recognize all the Chinese vegetables that she grows in there and have conversation with her. So it was very important that we found somebody that was culturally in line with us. […] So, one of the things that the Chinese people do, come September and April, is go visit the tombs of the passed/deceased and everything. So [worker] and I would put together this whole ceremony, we would take mom and we would go visit the graveyard – to the cemetery and perform all the food and everything, you know. And that’s part of it is that she still wants to do that. And with [worker] helping her in and out of the car, and helping me get all the food ready, it was wonderful. (Alberta, group 8)

The participants in the focus groups who were supporting clients with limited English ability chose the individual employer pathway as an opportunity to recruit and hire workers with shared linguistic and sometimes cultural backgrounds. The agency pathway was not seen as an option for meeting this goal.

### Theme five: Informal support networks

Finally, it emerged that DF home care, whether through an agency or the individual employer model, is predominantly possible only for those who have additional help from a co-residing spouse or family member, or extensive involvement from unpaid family and friends. Julia, who moved her mother into her home, described the network available to her mother: “There's four of us in the household so there's always someone that can be with grandma. But if we had to pay for those hours we'd never, ever have enough” (Manitoba, group 3). Bryce, who organized care for himself, spoke about his wife’s help and his extensive network of family and friends:

Bryce: [My wife] she does a large percentage of the work. I do have two adult children who help. But all of our parents are alive, and my wife’s mother in particular is a huge help to us. And then, I have siblings, we even have a neighbour who takes care of our driveway in the winter, which is amazing. And, yeah, a really nice network of family help. (Manitoba, group 1)

The focus groups took place during the pandemic, and some described how working at home enabled using DF home care:

Odell: [DF] home care is not enough for the full coverage of the needs of the person we're looking after. So the fact that we are, right now this year [my husband and I both work] at home we'll just see how well it works for us right now because we're home all the time, right? At night and during the day we don't travel so it's perfectly fine. The moment we start working [outside the home] we will see those huge gaps and it's not sufficient, no. And it's not enough respite for the people, for the caregivers, for us it's not enough break. As long as you – if you have a big family and your primary caregiver and some other family members gives you a break wonderful but we're alone we don't have any of that. (Manitoba, group 3)

Albert had moved for employment and was struggling to meet the care needs for his wife who was living with multiple sclerosis. Albert’s sleep, mental, and physical health were all impacted by the demands of care:

Albert: So I'm it. There is nobody else. […] It's not like I can call up a friend and say, hey, come over and help my wife do this or that. So it is a challenge, physically and mentally. I now have a bad back, I have a bad right shoulder. […] I'm on the plus side of 50 now and obviously my health is starting to be affected. More and more. And so my ability to do it. (Manitoba, group 5)

The importance of a robust informal support network was especially acute for those needing supervision and overnight care. Julia, who brought her mother to live with her, described her situation and the extent to which her family network pitched in to help:

Julia: And in our case like my mom could not have been left alone. So someone always had to be here, right? So I would – generally I could often times work my work schedule so that I could be here in the afternoon, my husband could be here in the evening when I had to be out at a board meeting or whatever. My brothers would come and stay for a weekend if we were going away for a weekend. My brother would fly in when we were going away on holidays that was his way of contributing. My two sons who are adults would often come and grandma sit because she couldn't be left alone. And so the seven hours a day never covered what we needed because she was 24 hour care, right? (Manitoba, group 3)

While perhaps not unique to DF home care, a solid, and perhaps extensive, informal support network is required to provide enough support for most people.

## Discussion

While acknowledging that DF home care is a niche option in Canada this study set out to shed light on the role of agency providers in DF home care in two Canadian provinces. There are advantages and disadvantages to including agencies in the DF home care landscape. Our findings add to existing and growing research that demonstrates clients and families are more satisfied with DF home care options [2, 4, 6]. The findings add that in the two differing provincial contexts in this study, satisfaction with home care services were improved through DF home care regardless of whether services were delivered through an agency or through individual employment. For many of the participants, experiences of DF were informed by experiences of other public services that the participants overwhelmingly found inadequate and inconsistent. This is an important finding, as it supports person-centred models of home care delivery that include high degrees of choice, consistency, and flexibility in contrast to regimented time-and-task based care. DF home care can demonstrate practices that may improve other public home care services in Canada. For example, “social task shifting,” where workers are allowed to take a more flexible and broader array of duties (e.g., help to attend social activities, a care plan that varies day to day) is a benefit of DF home care regardless of whether a person is using an agency or acting as an individual employer [37]. Experiences of people using DF home care programs provide a lens to critically reflect on what is working and what is not working within other home care delivery models in Canada.

Secondly, participants indicated that they purchased services from agencies primarily to reduce the administrative burden and stress of arranging DF home care as an individual employer. Administrative burden and paperwork are well documented as barriers to accessing DF home care in many countries [10]. There are serious health and social impacts of caregiver burden and burnout [38, 39], and any effort to reduce the work of system navigation and care coordination is important from a population health perspective [40]. While agencies might seem like an obvious solution to reducing the administrative burden of DF home care, participants in our study revealed economic barriers to accessing the support provided through agency care. Funding and finances are front and centre in the DF home care model, studies on DF home care can perhaps more easily reveal the extent to which people are adding their own money or topping up public services. As will be taken up by our team in a future publication, the study also found agencies are associated with lower rates of pay for workers.

The study adds to the limited literature that speculates on DF home care as an avenue to encourage linguistic and/or cultural matching between workers and those who need care. Like existing literature [13], we found that this can be a priority for some families. Our examples demonstrate that linguistic and cultural matching predominately takes place through the individual employer model, and further, all such cases among our participants had an English-speaking family member to organize the care. This suggests that, as in the UK-based study, there may be linguistic barriers for people with limited English language ability to accessing DF home care [12, 28]. Finally, as with regular home care options, clients with limited or inadequate informal support networks will struggle with the care available through this model. The vast majority of care at home is provided by unpaid caregivers [41], and this study confirms that families and clients using DF home care also rely on extensive additional support.

## Conclusions

DF home care in Canada, like other countries, is a key element of the public commitment to prioritize home care services that meet the needs of diverse older people and people with disabilities. Yet, as these programs expand in size and popularity, it is essential to reflect on the barriers that can be created through decisions in policy design. In regions that allow for clients and families to apply DF home care through home care agencies, for example, it may inadvertently create barriers for low-income individuals, those with limited social supports, and clients with limited English. DF home care is regarded by many as a ‘better” option with higher satisfaction consistently reported by clients; as such, the shortfalls and barriers must be confronted *within* DF programs in Canada. Given the growing inequalities that exist in Canadian society, the best form of public home care needs to be made available to all who need it, irrespective of ability to pay, degree of social support, or competence in the English language. That is, DF home care must not become an elite version of public services that is only afforded to some.

### Limitations

The study took place in two specific cities and provinces; our thematic coding structure was sustained in both settings revealing similar themes and sub-themes. The programs both allow for agency use and as such, the results may not apply to programs in different contexts. We do not have information about the participants who chose not to participate in the study and there may be participant bias in the findings.

## Supplementary Information

**Additional file 1.**

## Data Availability

The datasets generated during and analyzed during the current study are not publicly available due to the possibility of compromising individual privacy but may be made available by the corresponding author on reasonable request.

## Abbreviation

DF: Directly-funded home care

## Footnotes

1 There are numerous terms that vary regionally and sometimes more broadly include individualized or personalized care plans. In this article, we focus on programs that include a cash transfer or budget for care. Other terms include but are not limited to: cash and counselling; cash-for-care; direct payments; consumer directed care; and self-managed care.

2 As mentioned above, home care services vary in each province; however, this article focuses on common themes across the two study sites. Of note, we found very limited provincial variation in the findings of this study, with the exception of rates of funding likely due to differences in local economies and an observation that some clients in Alberta were using the funds to support a live-in caregiver.
