# Supplementary material for: Inequities in access to directly-funded home care in Canada: a privilege only afforded to some
Source: BMC Health Serv Res. 2023 Jan 18;23:51. doi: 10.1186/s12913-023-09048-9 (PMC9847438; doi:10.1186/s12913-023-09048-9)
Supplement: Supplementary file 1 — Additional file 1. [file 12913_2023_9048_MOESM1_ESM.docx]

**Focus Group Guide for Clients and Families – Agency Hire**

# Experiences/background

1. We can open by going around the group and saying your name and why you chose to use self-managed care?
2. In broad strokes, what kind of things do your workers help with? What we are trying to get at with this question is if your workers can do things that not all workers can do, such as help with shopping, or go on walks, or provide companionship.
   - Is there anything the workers are not allowed or are unwilling to do? (***Prompt:*** dog walking, shopping, laundry, you can give an example)
3. Do other people (friends, family members, or private support) also help out? (***Prompt:*** *Specifics)*
4. In what ways has COVID-19 affected your services? (***Prompt*:** service disruptions, feelings of risk)

# Home care agencies, opinions/perspectives

1. We are comparing the experiences of those who hire workers from their own networks with self and family managers who use home care agencies. **Not all self-managed care programs in Canada allow people hire agencies!** Can you tell me how you came to use agency services?

**Prompt:** How did you find out about the agency? Did you compare multiple agencies before selecting this one? What went into your decision?

1. Does anyone in the room have experiences with both hiring directly and using an agency?
2. Do you **supplement your care with personal funds**? *(****Prompts:*** *Administrative costs, food for workers, other)*
3. Io you that think it is important to have some extra financial resources if you use some form of family managed care?
4. What do you think are the disadvantages and advantages of using an agency vs hiring directly? *(****Prompt:*** *Control, better scheduling, choice of characteristics of worker, other?)*
5. One of the advantages of self-managed care is the ability to find workers with a shared linguistic, cultural or other important background (e.g, speaking the same language, practicing the same religion). Does anyone have experiences with that that you would like to share?
   - Home care workers can include people from a variety of cultural, linguistic, and religious backgrounds, and many are also new Canadians and immigrants. How do you navigate cultural differences between you or you and your family member and your workers?
6. Have you ever had a conflict with your workers? How was it resolved?
   - Does the agency help or hinder the situation? How?
7. Going a step further, have you ever been harmed by a worker (for example, financial, physical harm, sexual, and/or verbal abuse). (may prompt: you do not have to answer this if people look uncomfortable)
   - If yes, how did you handle it?

# Working conditions and intersectionality

1. Are you involved in selecting your workers?
2. Does gender play a role in your decision to hire a worker? Are you comfortable with a [specific gender] helping you?

# Additional question for rural participants;

1. Do your workers have to travel long distances to help you? What happens if the weather is bad or your worker is sick?
2. Some of you live in an area you would describe as “rural.” Can you speak to some of the benefits and challenges in living in rural areas related to using Self and Family Managed care?

# Closing

1. What advice would you give somebody who is thinking about using self-managed home care? Hiring through an agency?

**Thank you so much for your time.** Before we end, is there anything else you would like to talk about?
